# Polarity-Dependent Misperception of Subjective Visual Vertical during and after Transcranial Direct Current Stimulation (tDCS)

**DOI:** 10.1371/journal.pone.0152331

**Published:** 2016-03-31

**Authors:** Taiza E. G. Santos-Pontelli, Brunna P. Rimoli, Diandra B. Favoretto, Suleimy C. Mazin, Dennis Q. Truong, Joao P. Leite, Octavio M. Pontes-Neto, Suzanne R. Babyar, Michael Reding, Marom Bikson, Dylan J. Edwards

**Affiliations:** 1 Department of Neuroscience and Behavioral Sciences, Ribeirao Preto Medical School, University of Sao Paulo, Ribeirao Preto, SP, Brazil; 2 Neural Engineering Laboratory, Department of Biomedical Engineering, The City College of New York of the City University of New York, New York, New York, United States of America; 3 Non-invasive Brain Stimulation and Human Motor Control Laboratory, Burke Medical Research Institute, White Plains, New York, United States of America; Neurology Department, Weill Medical College, Cornell University, New York, New York, United States of America; University Medical Center Goettingen, GERMANY

## Abstract

Pathologic tilt of subjective visual vertical (SVV) frequently has adverse functional consequences for patients with stroke and vestibular disorders. Repetitive transcranial magnetic stimulation (rTMS) of the supramarginal gyrus can produce a transitory tilt on SVV in healthy subjects. However, the effect of transcranial direct current stimulation (tDCS) on SVV has never been systematically studied. We investigated whether bilateral tDCS over the temporal-parietal region could result in both online and offline SVV misperception in healthy subjects. In a randomized, sham-controlled, single-blind crossover pilot study, thirteen healthy subjects performed tests of SVV before, during and after the tDCS applied over the temporal-parietal region in three conditions used on different days: right anode/left cathode; right cathode/left anode; and sham. Subjects were blind to the tDCS conditions. Montage-specific current flow patterns were investigated using computational models. SVV was significantly displaced towards the anode during both active stimulation conditions when compared to sham condition. Immediately after both active conditions, there were rebound effects. Longer lasting after-effects towards the anode occurred only in the right cathode/left anode condition. Current flow models predicted the stimulation of temporal-parietal regions under the electrodes and deep clusters in the posterior limb of the internal capsule. The present findings indicate that tDCS over the temporal-parietal region can significantly alter human SVV perception. This tDCS approach may be a potential clinical tool for the treatment of SVV misperception in neurological patients.

## Introduction

Subjective visual vertical (SVV) perception measures one’s ability to align their sense of vertical with true ‘Earth vertical’ [1]. SVV testing is a widely used modality to assess vertical perception and is considered the most sensitive sign of vestibular imbalance in the roll plane [2]. Pathologic misperceptions of SVV are frequent in patients with stroke and vestibular disorders, and have been strongly correlated with functional disability in these patients [3–7]. Effective strategies to improve SVV misperceptions do not yet exist.

Transcranial direct current stimulation (tDCS), transcranial magnetic stimulation (TMS) and galvanic vestibular stimulation (GVS) are non-invasive methods for modulating cortical excitability that have the potential to influence different brain functions depending on the stimulated region and specific techniques used [8]. Non-invasive neuromodulation techniques in vestibular research have been recently reviewed [9]. The vestibular ocular reflex (VOR) and movement perception during rotational stimulation are significantly altered by tDCS [10]. Cathodal stimulation over left posterior parietal cortex has been shown to induce modulation of the VOR [9, 11]. In addition, GVS can alter haptic and visual vertical perceptions in healthy subjects and stroke patients [12, 13].

Composite analysis of brain lesions and functional imaging techniques indicate that the parietal cortex, posterior temporal cortex, insula, and temporal-parietal junction are multimodal areas involved with accurate perception of SVV [14–16]. Most recently, repetitive TMS (rTMS) over a small region of the supramarginal gyrus was found to produce a transitory SVV tilt in healthy subjects [17]. Analyzing the effect of tDCS on SVV in healthy subjects is the next logical step because tDCS costs less and has greater portability, safety and ease of use than rTMS [18]. These attributes of tDCS make it a practical modality to potentially correct altered SVV of patients in a clinical setting. However, it has not been previously studied.

In the present study, we investigated whether bipolar-balanced tDCS over the temporal-parietal region could produce a tilting effect on the SVV in healthy subjects. We also studied the effect of tDCS polarity on the direction of SVV tilt, and whether an after-effect existed in SVV tilt following tDCS application. SVV misperception towards the contralesional side after stroke has been reported [6]. Hypothetically, based on findings from interhemispheric balance between the motor cortices [19, 20] or between cortical regions related to visuospatial perception [21], increased interhemispheric inhibitory drive from the unlesioned to the lesioned cortex creates this SVV misperception observed in stroke patients. Transcranial direct current stimulation has the ability to induce an imbalance between cortical sites because the anode generally upregulates excitability of underlying brain regions while the areas under the cathode are down-regulated [22]. Thus, we predicted that tDCS would create an interhemispheric imbalance between cortices that would induce SVV tilts toward the anode in healthy subjects [23]. Such ability to selectively regulate brain regions may prove useful in counteracting SVV misperceptions after stroke.

## Materials and Methods

### Participants

We studied thirteen right-handed healthy subjects (6 men, 7 women; mean age 29.6 ± 3.8 years) with no evidence of brain, vestibular or orthopedic dysfunction. Additional oculomotor tests, head shake test, and head thrust test were performed to guarantee the exclusion of vestibular deficits [24]. All participants had normal or corrected-to-normal vision. They were naïve and blind to the tDCS approach and the study purpose.

This study was conducted according to the Helsinki Declaration requirements for human investigation, and was approved by the local ethics committee. All participants provided written informed consent.

### Stimulation Protocol

Bipolar-balanced tDCS [25] was delivered through two saline-dampened electrodes of circular shape (diameter of 5 cm) held in place by a neoprene EEG cap. Electrodes were placed at the circumcenter of a triangle with vertices on C3, T3, P3 over the left hemisphere and C4, T4, P4 over the right hemisphere using EEG 10/20 coordinates. Stimulation sites were selected to optimize coverage of the supramarginal gyrus, temporal-parietal junction area and the parietal insular vestibular cortex (PIVC) [26]. Three experimental sessions (Sham, Right-anode/Left-cathode, Left-anode/Right-cathode) were performed randomly on different days with an interval of at least 24 hours. A battery-driven constant current stimulator (Chattanooga^®^ Ionto, USA) was set to deliver 2mA for 20 minutes. The device provided an automatic 30-second current ramp up and down during power on/off to keep participants comfortable. In the sham control condition the position of the anode (right versus left hemisphere) was randomly chosen; ramp up (30 sec) was identical to the ‘real’ bipolar-balanced stimulation, but then the device was immediately ramped down over 30-seconds (total time of stimulation = 60 seconds). Participants maintained their eyes closed during stimulation, except when they were performing the SVV tasks to systematize the visual vertical testing [27] and to improve subjects’ blindness. In order to assure that subjects were blind to the montage, they were asked whether they could perceive differences regarding stimulation protocols after the three experimental sessions.

### Test

**Fig 1:**
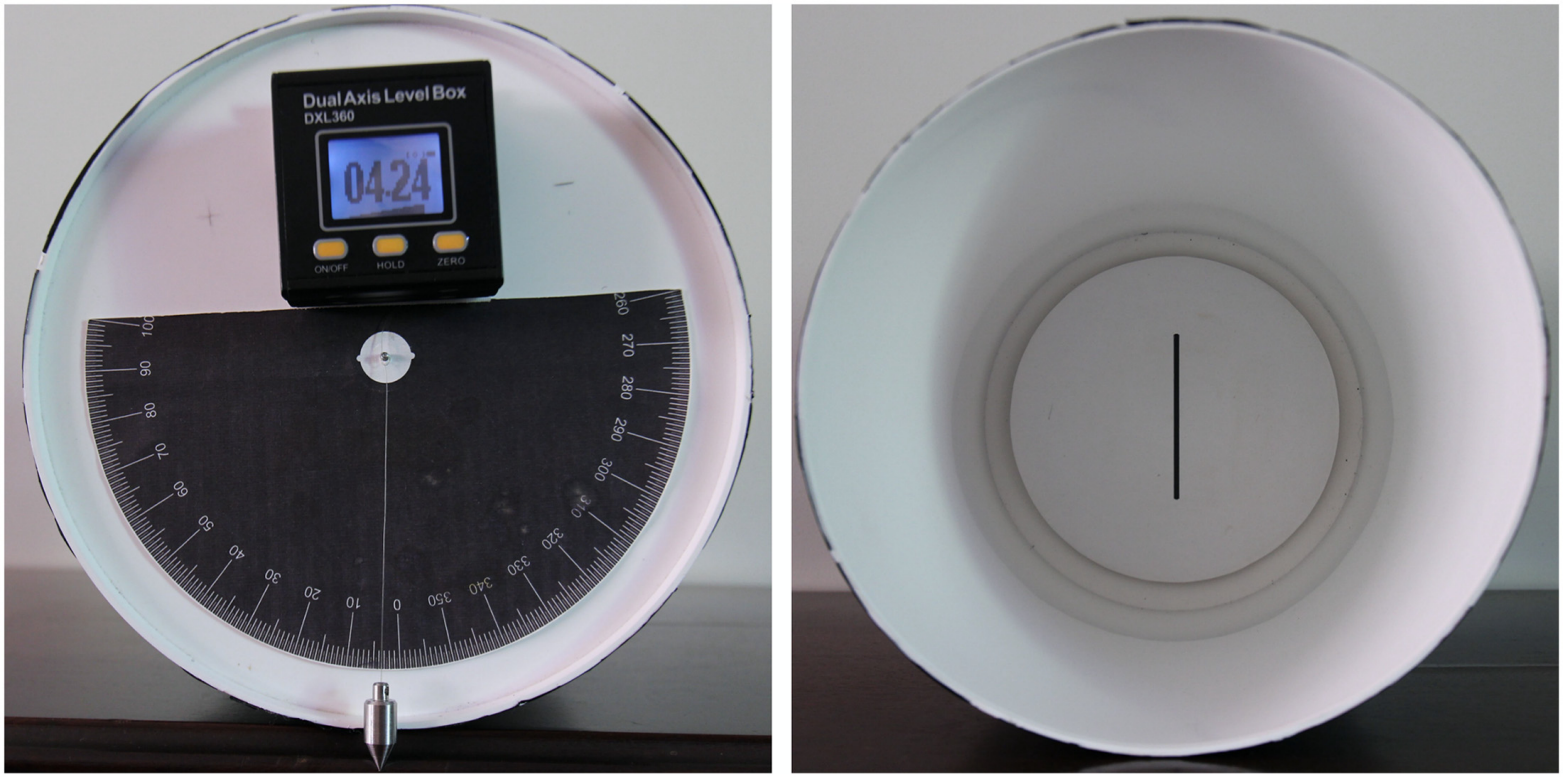
“Bucket method” for measuring Subjective Visual Vertical. Note the exterior digital inclinometer (left) and interior line reference.

SVV was determined using the "bucket method" [28]. To perform the assessments, each participant remained seated upright in a chair with back and foot support and with their trunk restrained by bands. Participants visualized a black line (10.5 cm long, 0.4 cm wide, at 25 cm distance) inside the bottom of the bucket. On the exterior bottom of the bucket, a protractor was aligned perpendicular to the dark line inside with a pendulum suspended from the axis of bucket rotation. This was used to calibrate a digital inclinometer with a precision of 0.01° (Fig 1) before each session. A positive sign indicated clockwise SVV tilt and a negative sign a counterclockwise SVV tilt. Participants looked into the bucket while the examiner manually rotated it slowly in clockwise (+SVV) and counterclockwise (-SVV) directions. Subjects verbally reported when the line inside the bucket appeared upright. One examiner was blinded to the inclinometer measurements. This examiner also observed and supervised the position of the participant’s head. A second examiner registered the SVV results. All subjects practiced at least six trials to exclude a learning effect and were instructed to make as many corrections as they needed to set the line in their perceived vertical. Each SVV assessment consisted of 10 trials, 5 beginning with the upper edge of the line in the clockwise and 5 in the counterclockwise tilt in a random order and angulation. SVV was examined at several time-points in relation to tDCS intervention: before tDCS (T0: baseline), during tDCS (T1: 30 seconds after the start of stimulation, T2: 15 minutes after the beginning of stimulation); and after tDCS (T3: immediately after, T4: 15 minutes after the end of stimulation, T5: 30 minutes after stimulation).

### Modeling of the tDCS induced current flow

Brain stimulation as represented by cortical electric field and current density was predicted during tDCS by modeling electrostatic physics with Finite Element (FE) models. Three-dimensional head models consisting of tissues with varying material properties (conductivities) were created using subject-specific MRI data. MRI scans (1mm^3^ resolution) of two subjects were previously segmented into 7 materials of varying conductivity (S/m): skin (0.465), fat (0.025), bone (0.01), cerebrospinal fluid or CSF (1.6), grey matter (0.276), white matter (0.126), and air (10^−15^) [29]. This parameter set was named as “Standard”. Subject ‘S0’ is a reference model of an adult male. Subject ‘S4’ is a model of an adult female. Both subjects were previously found to represent a relatively wide variance in the cortical electric field [30]. An alternative set of parameters used by other groups [31] was also modeled to compare the sensitivity of the predictions. This alternative set was named “H1”. These alternate conductivities (S/m) were: skin & fat (0.08), bone (0.013), CSF (1.8), and grey & white matter (0.1). Circular sponges and electrodes (50mm diameter x 5mm thickness) were modeled with conductivities of 1.4 and 5.99x10^7^ S/m respectively. Adaptive volumetric meshes were generated for each subject and montage using ScanIP (Simpleware, Exeter, UK). Volume conductor physics were applied in an FE package (COMSOL, Burlington, MA) and voltage was solved for using the following boundary conditions: 2 mA inward current on the anode surface, ground on the cathode, and insulation on the remaining exposed surfaces. Cortical electric field magnitude was calculated to represent stimulation. In order to visualize three-dimensional current flow, streamlines that trace current density direction were seeded on the anode-sponge boundaries with streamline radius proportional to the log of current density magnitude.

### Sample size

According to Cocks and Torgerson [32], a pilot study requires at least 9% of the sample size calculated to the final trial. A sample size of 13 participants corresponds to 10% of the estimated a sample size for the final trial, considering an effect size of 0.25, assuming 80% power, alpha of 5% and a two-sided paired t-test.

### Statistical analyses

This study is a randomized, sham-controlled, single blind crossover pilot study. The subjects and the statistician who performed the analyses were blind to the condition settings. Descriptive analyses of the data consisted of means and standard deviations for each time and each condition.

Data were analyzed using a linear mixed-effect model, according to statistical guidelines for the analysis of crossover studies [33]. SVV degree was considered as the outcome variable, and the subject’s effect was considered as the random effect to explain the correlation between repeated measures of the same subject. The time (T0 to T6) and type of condition (Sham, R-anode/L-cathode, L-anode/R-cathode) and the sequence of each condition were considered as the fixed effects. The comparison among means was made through the orthogonal contrasts. The hypotheses involved in the contrasts were tested using the t-test. Since the hypotheses were defined a priori, no adjustments for multiple comparisons were performed [34]. The two-sided testing hypothesis was that there would be a difference between sham condition and active condition among each time-point of assessment, and between baseline time-point and the after-stimulation time-points among stimulation conditions.

The assumption of normality of the residuals and homoscedasticity of the variances were investigated by the normal plot and dispersion graphs between the residuals and the observed values. In all tests, a 5% level of significance was used (two-sided). Statistical analyzes were performed using the SAS System version 9.2 (SAS Institute Inc., Cary, NC, USA) and the graphs were built using the R Project for Statistical Computing.

The calculation of effect size between tDCS conditions in each time-point was performed by

$$Cohen\prime s\;d=\frac{{\bar{x}}_{1}-{\bar{x}}_{2}}{s_{pooled}},$$

where ${\bar{x}}_{1}$ and ${\bar{x}}_{2}$ are the sample means of the tDCS conditions, and *s*_*pooled*_ was estimated by

$$s_{pooled}=\sqrt{\frac{s_{1}^{2}\left(n_{1}-1\right)+s_{2}^{2}\left(n_{2}-1\right)}{n_{1}+n_{2}-2}}$$

where $s_{1}^{2}$, $s_{2}^{2}$, *n*_1_ and *n*_2_ are the respective variance and sample size of the tDCS conditions. The effect size between time-points of SVV assessment was calculated by dividing the mean estimated difference between baseline and the other time-points by the SD for each tDCS conditions [35]. Effect size was considered small when d = 0.2, medium when d = 0.5, and large when d = 0.8 [36].

## Results

The descriptive statistics are shown in Table 1. No significant differences were observed in the baseline (T0) performances among conditions. In the comparisons with sham condition at T1 and T2, there was small effect in the condition R-anode/L-cathode and large effect in the condition L-anode/R-cathode (Table 1). During stimulation, condition Right-anode/Left-cathode induced clockwise SVV tilts and condition Left-anode/Right-cathode induced counterclockwise tilts. The comparison between sham and active conditions revealed rebound effects immediately after both active conditions.

**Table 1:** Descriptive data of SVV for each time and condition, and comparisons between pairs of conditions for time-points of SVV assessment.

Label	Mean (SD)	Mean (SD)	Mean (SD)	Estimate	p-value	Lower _95%_CI	Upper _95%_CI	Effect Size
Time-points of SVV assessment	Sham	R-anode/ L-cathode	L-anode/ R-cathode	Comparison between Sham and R-anode/L-cathode				
**T0**	-0.07 (1.12)	0.01 (1.18)		-0.08	0.572	-0.35	0.19	-0.07
**T1**	-0.22 (1.01)	0.64 (1.32)		-0.86	**<0.0001**	-1.13	0.59	**-0.70**
**T2**	-0.13 (1.15)	0.52 (1.32)		-0.65	**<0.0001**	-0.92	0.38	**-0.50**
**T3**	-0.16 (1.08)	-0.50 (1.45)		0.34	**0.013**	0.07	0.61	**0.25**
**T4**	-0.19 (1.33)	-0.02 (1.32)		-0.28	**0.050**	-0.55	0.00	-0.12
**T5**	-0.25 (1.01)	-0.06 (1.24)		-0.19	0.168	-0.46	0.08	-0.16
				**Comparison between Sham and L-anode/R-cathode**				
**T0**	-0.07 (1.12)		0.10 (1.50)	-0.17	0.216	-0.44	0.10	-0.12
**T1**	-0.22 (1.01)		-0.91(1.64)	0.690	**<0.0001**	0.42	1.96	**0.48**
**T2**	-0.13 (1.15)		-0.81 (1.68)	0.68	**<0.0001**	0.40	0.95	**0.45**
**T3**	-0.16 (1.08)		0.14 (1.73)	-0.30	**0.029**	-0.57	-0.03	**-0.20**
**T4**	-0.19 (1.33)		-0.21 (1.5)	0.02	0.878	-0.25	0.29	0.01
**T5**	-0.25 (1.01)		-0.40 (1.53)	0.15	0.267	-0.12	0.42	0.11
				**Comparison between R-anode/L-cathode and L-anode/R-cathode**				
**T0**		0.01 (1.18)	0.10 (1.50)	-0.09	0.502	-0.36	0.18	-0.06
**T1**		0.64 (1.32)	-0.91(1.64)	1.55	**<0.0001**	1.28	1.82	**1**
**T2**		0.52 (1.32)	-0.81 (1.68)	1.33	**<0.0001**	1.06	1.60	**0.84**
**T3**		-0.50 (1.45)	0.14 (1.73)	-0.64	**<0.0001**	-0.91	-0.37	**-0.38**
**T4**		-0.02 (1.32)	-0.21 (1.5)	0.30	**0.035**	0.02	0.57	0.13
**T5**		-0.06 (1.24)	-0.40 (1.53)	0.34	**0.013**	0.07	0.61	**0.23**

SVV = subjective visual vertical; SD = standard deviation; R = right; L = left; _**95%**_CI = 95% confidence interval of the difference between means (effect size).

Except for baseline performance, there were statistically significant differences in all times in the comparison between active treatment conditions R-anode/L-cathode and L-anode/R-cathode (Table 1).

Regarding the comparisons of SVV among times for each tDCS condition, there were significant differences of T0 with T1 and T2 with small effect in the conditions R-anode/L-cathode and medium effect in condition L-anode/R-cathode (Table 2). The immediate after-effect showed a significant difference at T0 and T3 in the R-anode/L-cathode condition only. Lasting after-effects were observed only in the condition L-anode/R-cathode since there were significant differences of T0 with T4 and T5 (Table 2). The after-effects in T4 and T5 were also significantly different when the conditions R-anode/L-cathode and L-anode/R-cathode were compared. The effect sizes of each comparison are shown in Tables 1 and 2. All data are freely available in S1 Table for researchers to use, whenever this is legal and ethical. SAS procedures for statistical analysis are in S1 Text and graphs of each stimulation condition showing the mean and standard error of SVV scores of each subject are in S1, S2 and S3 Figs.

**Table 2:** Comparisons between time-points of SVV assessment for the tDCS conditions.

Time-points of SVV assessment	Estimate	p-value	Lower _95%_CI	Upper _95%_CI	Effect Size
***Sham Condition***					
**T0–T1**	0.15	0.266	-0.12	0.42	0.13
**T0–T2**	-0.07	0.62	-0.20	0.33	0.05
**T0–T3**	0.09	0.51	-0.18	0.36	0.07
**T0–T4**	0.15	0.36	-0.15	0.39	0.08
**T0–T5**	0.18	0.182	-0.09	0.45	0.12
***R-anode/L-cathode Condition***					
**T0–T1**	-0.63	**<0.0001**	-0.90	-0.36	**-0.44**
**T0–T2**	-0.50	**0.0003**	-0.77	-0.23	**-0.34**
**T0–T3**	0.51	**0.0002**	0.24	0.78	**0.34**
**T0–T4**	-0.07	0.602	-0.35	0.20	0.02
**T0–T5**	0.07	0.60	-0.20	0.34	0.05
***L-anode/R-cathode Condition***					
**T0–T1**	1.01	**<0.0001**	0.74	1.28	**0.65**
**T0–T2**	0.92	**<0.0001**	0.65	1.18	**0.54**
**T0–T3**	-0.04	0.781	-0.31	0.23	-0.02
**T0–T4**	0.32	**0.022**	0.05	0.59	0.18
**T0–T5**	0.51	**0.0002**	0.24	0.78	**0.35**

SVV = subjective visual vertical; R = right; L = left; _**95%**_CI = 95% confidence interval of the difference between means.

**Fig 2:**
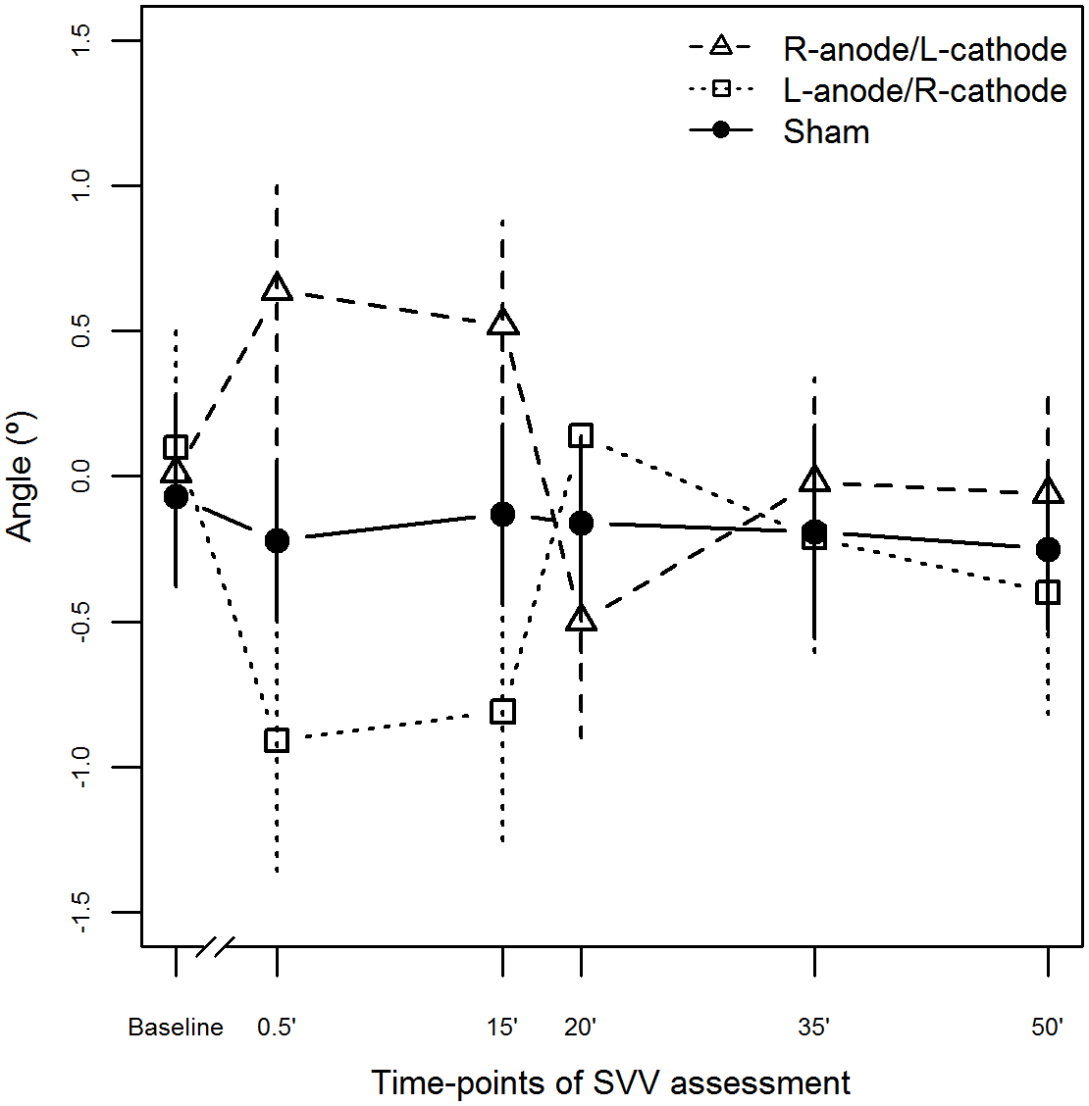
Mean and standard error of SVV scores for tDCS conditions at different times. Before tDCS (T0 = Baseline), during tDCS (T1 = 0.5min: 30 seconds after the start of stimulation; T2 = 15min: 15 minutes after the beginning of stimulation); and after tDCS (T3 = 20min: immediately after; T4 = 35min: 15 minutes after the end of stimulation; T5 = 50min: 30 minutes after the stimulation).

**Fig 3:**
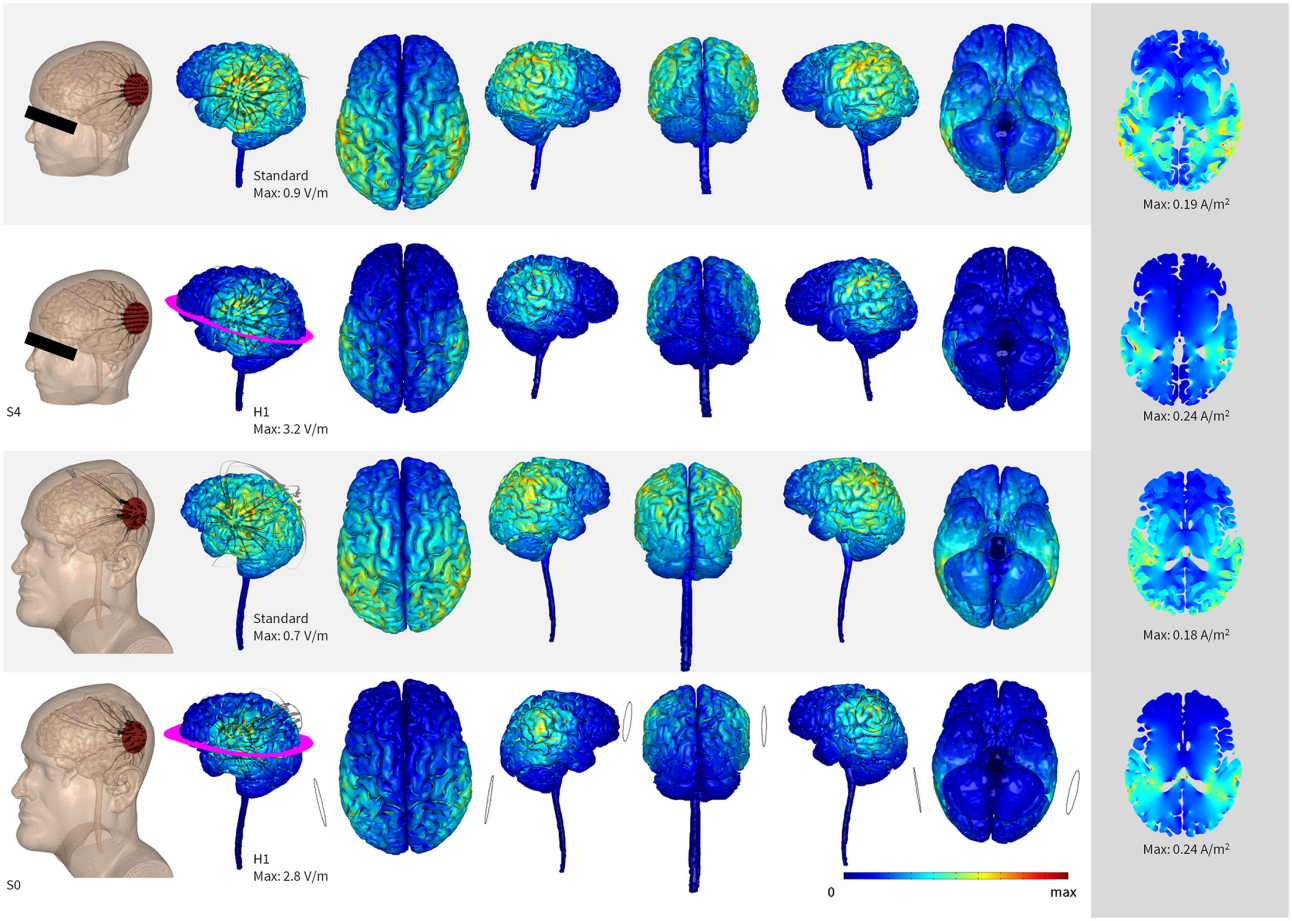
Sensitivity analysis of vertical perception tDCS using Finite Element Analysis. Bilateral temporal-parietal region stimulation with 5cm diameter electrodes was modeled in “two subjects,” each using two different conductivity sets, “Standard” and “H1”. Electric field (V/m) and current density (A/m^2^) were predicted for 2mA of stimulation. Columns 1 and 2 demonstrate the relative electrode position with current streamlines, whose radii are proportional to the logarithm of current density. The magenta ring represents the location of the axial slice in the far right column. Simulations using standard conductivity values resulted in diffuse electric field throughout the parietal lobe, while H1 conductivity values resulted in more concentrated cortical stimulation. Across two head models and two conductivity sets, the most reliable cortical and subcortical regions of influence were under the electrodes.

Fig 2 shows the mean SVV results of all conditions at different times. The mean duration of each set of 10 SVV tests was 3.58 ± 0.8 minutes. After the end of the 3 sessions, all the participants reported no perceived qualitative difference among conditions demonstrating that they were blind to the stimulation protocol and the sham method was effective. Current flow models predicted the stimulation of temporal-parietal regions under the electrodes and deep clusters in the posterior limb of the internal capsule (thalamic radiation) (Fig 3).

## Discussion

This study provides the first evidence that bipolar-balanced tDCS conditions applied over the temporal-parietal region have both online effects and after-effects on visual vertical perception. There were clear and tilted SVV towards the anode for both ‘real’ stimulation conditions that confirm tDCS neuromodulation of this perception. The lack of effect of sham stimulation confirms that the differences found in both active tDCS conditions were true treatment effects. The small magnitude of SVV change reported here could also be attributed to the usage of a single stimulation exposure to each tDCS condition. Other approaches such as high-definition tDCS (HD-tDCS) and random noise stimulation have the potential to produce even greater effects [37, 38].

Bipolar-balanced tDCS as applied in this protocol over the posterior parts of the temporal-parietal cortex targeted brain regions most frequently associated with vertical misperceptions in studies based on lesion analysis [39–44]. Other evidence supporting the relevance of these regions to accurate assessment of SVV comes from high-density EEG recordings of brain activity during visual vertical performance tasks. These studies described early-evoked potentials localized in right lateral temporal-occipital cortex and later evoked potentials localized in bilateral temporal-occipital and parietal-occipital cortical regions [16]. Recently, continuous theta burst rTMS over the posterior part of the right supramarginal gyrus was found to tilt vertical perception in healthy subjects [17]. Combining lesion analysis methods, functional techniques, and neuromodulation approaches thus strengthens ones confidence in the validity of our observations [45].

A previous study has analyzed vestibular ocular reflex (VOR) and rotation perception during rotational stimulation in healthy subjects [10]. They studied VOR responses during and after bipolar-balanced tDCS via electrodes placed over CP6 and CP5, approximately 1 cm below of the electrode localization used in the present study. They found an increase in VOR and rotational perceptual thresholds during tDCS and approximately 2 minutes after tDCS. However, they did not find a polarized effect based on the side of anodal stimulation and both active conditions increased VOR and perceptual thresholds [10]. In this context, after continuous theta burst TMS (cTBS) over the posterior part of the right supramarginal gyrus, Kheradmand et al. [17] found visual vertical tilts towards the stimulated hemisphere, i.e., towards the region that had its excitability decreased [46], in one subject whose head was in the upright position. However, when the other 8 subjects were assessed with their heads tilted 20°, SVV was always tilted opposite to the direction of the head tilt after cTBS [17]. Here we evaluated subjects with head in the upright position and found a SVV tilt towards the anode and away from the cathode, i.e., towards the hemisphere that had its excitability increased and away from the hemisphere that had its excitability decreased [37]. These opposite outcomes might be related to the chosen parameters and type of stimulation because tDCS has more diffuse neuromodulation effects than rTMS [for review [37, 46]. Also, the side of stimulation may explain the opposite outcomes because rTMS was applied in the right hemisphere and tDCS was applied bilaterally [1, 10, 17, 47]. Alternatively, the directional specificity of neuronal excitability might have been changed due to the relatively high intensity (2mA) and longer duration (20 min) used in the present study. There is also evidence of reversed motor cortical excitability after the same parameters of tDCS [48].

Sponge pad tDCS may not support detailed anatomical targeting [49]. We therefore investigated current distribution under the tDCS montages used in our protocol. Even considering possible variability of current flow during tDCS between subjects [49] as shown in Fig 3, the induced electrical fields model indicated clusters of activation in most areas of the neural network that processes perceptions of verticality: superior temporal sulcus, temporal-parietal junction, supramarginal gyrus, and insular cortex (Fig 3)[16, 17, 39, 43]. Also, deep current clusters were found in the posterior limb of the internal capsule (thalamic radiation), which connect the thalamic nuclei involved in vestibular processing (Fig 3)[50]. Therefore, the physics of our current flow analysis add further support to our study hypothesis.

The underlying mechanism of SVV misperception induced by tDCS in healthy subjects might involve interhemispheric mis-balance produced by decreased excitability of cerebral regions under the cathode and increased excitability under the anode as occurs with visuospatial perception [23]. Polarized SVV effects after tDCS may indicate an interhemispheric interaction within the verticality perception network. Polarity-specific effects may be influenced by neuronal network architecture of specific temporal-parietal regions [51]. Assessing SVV is a complex sensory, perceptual, cognitive task. We analyzed only one of the possible effects of tDCS over this multimodal region. Other brain functions were probably also affected and warrant future studies [52].

One may question whether the tDCS current used in this protocol also modulated the peripheral vestibular system resulting in SVV effects that were due to galvanic vestibular stimulation (GVS) as opposed to cortical stimulation. GVS can produce vestibular ocular torsion that leads to SVV tilt towards the anode [12, 13, 53, 54]. Moreover, the torsional response is present with GVS currents as low as 0.3 mA when electrodes are placed on the mastoids [55]. Computational models show that tDCS over the posterior parietal cortex produces electric fields in the inferior temporal gyrus and behavioral changes consistent with parietal neuromodulation [56, 57]. Current modeling also shows diffuse current flow through the skin and CSF, as well as the brain [58]. Across individuals and model assumptions, current is consistently predicted in the parietal regions under the electrodes. Outside the brain, current was not concentrated either in the ear canals or the pneumatized temporal bone (where the vestibular receptors are located). While tDCS of the peripheral vestibular system—or for that matter cranial nerves or other brain regions—cannot be excluded, our current modeling and behavioral result support our parietal cortical stimulation hypothesis. These conclusions are in agreement with the observations of Kyriakareli and coworkers after posterior parietal tDCS [10].

Immediately after tDCS, we observed a tilting effect towards the cathode in both active stimulation conditions. Several evidences indicate that both anodal and cathodal tDCS mainly affect resting membrane potential during stimulation (for review see [59]). We hypothesize that the opposite SVV tilt observed immediately after tDCS in relation to the online effect might be related to homeostatic mechanisms which aim to preserve plastic changes within a physiologically useful range and allows network stability [59, 60]. In this scenario, a sustained modulated membrane state produced by the constant current delivered by the protocol used in the present study might react to the stoppage of the stimulation by reversing its state and, thus, transiently producing an opposite effect.

There was no difference among time-points T4 and T5 between the sham-condition and active-conditions, however, relative to baseline the after-stimulation time-points among condition left anode/right cathode stimulation showed a small but significant tDCS after-effects up to 30 minutes after the end of stimulation. We speculate that the presence of a long-lasting effect of tilted SVV towards the same direction observed during the stimulation might have been related to the dominance for visual vertical cortical function in the non-dominant hemisphere [16, 26]. Moreover, long-lasting after-effects have been associated with neuroplasticity mechanisms such as long-term potentiation and depression, which are thought to underlie memory and learning [59]. Therefore, repetitive tDCS sessions might produce longer-lasting SVV effects.

Two possible limitations of the present study could be the simple methodology of SVV assessment and the lack of experimenter blinding. However, the SVV assessment was not influenced by experimenter bias. Moreover, the “bucket method” was already validated with accuracy and reliability as good as those of more sophisticated methods [28].

In conclusion, the present findings indicate that bipolar-balanced tDCS over the temporal-parietal region can produce a tilting effect on SVV in healthy subjects and can last for 30 minutes. Future studies are required to determine optimal stimulation parameters for a longer lasting and more robust SVV effect and clinical application for patients with vertical perception disorders.

## Supporting Information

S1 Fig Mean and standard error of SVV scores of each subject for sham condition at different times. Before tDCS (T0 = Baseline), during tDCS (T1 = 0.5min: 30 seconds after the start of stimulation; T2 = 15min: 15 minutes after the beginning of stimulation); and after tDCS (T3 = 20min: immediately after; T4 = 35min: 15 minutes after the end of stimulation; T5 = 50min: 30 minutes after the stimulation).
(TIF)

S2 Fig Mean and standard error of SVV scores of each subject for tDCS condition Right anode/Left cathode at different times. Before tDCS (T0 = Baseline), during tDCS (T1 = 0.5min: 30 seconds after the start of stimulation; T2 = 15min: 15 minutes after the beginning of stimulation); and after tDCS (T3 = 20min: immediately after; T4 = 35min: 15 minutes after the end of stimulation; T5 = 50min: 30 minutes after the stimulation).
(TIF)

S3 Fig Mean and standard error of SVV scores of each subject for tDCS condition Left anode/Right cathode at different times. Before tDCS (T0 = Baseline), during tDCS (T1 = 0.5min: 30 seconds after the start of stimulation; T2 = 15min: 15 minutes after the beginning of stimulation); and after tDCS (T3 = 20min: immediately after; T4 = 35min: 15 minutes after the end of stimulation; T5 = 50min: 30 minutes after the stimulation).
(TIF)

S1 Table Dataset. (XLS)

S1 Text SAS procedures for statistical analysis. (PDF)
