# Supplementary figures and images for: Polarity-Dependent Misperception of Subjective Visual Vertical during and after Transcranial Direct Current Stimulation (tDCS)
Source: PLoS One. 2016 Mar 31;11(3):e0152331. doi: 10.1371/journal.pone.0152331 (PMC4816520; doi:10.1371/journal.pone.0152331)

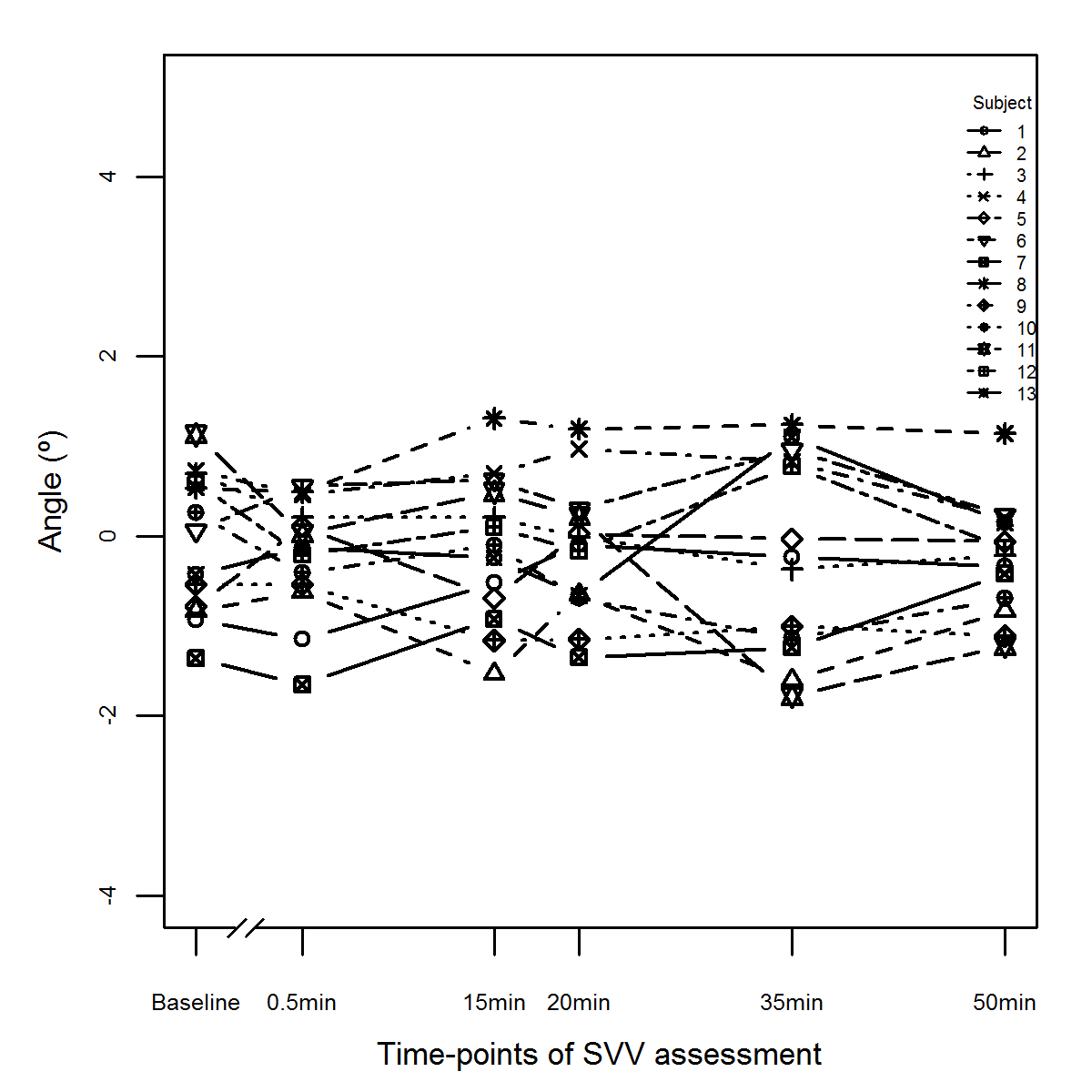

Supplement: S1 Fig — Before tDCS (T0 = Baseline), during tDCS (T1 = 0.5min: 30 seconds after the start of stimulation; T2 = 15min: 15 minutes after the beginning of stimulation); and after tDCS (T3 = 20min: immediately after; T4 = 35min: 15 minutes after the end of stimulation; T5 = 50min: 30 minutes after the stimulation). (TIF) [file pone.0152331.s001.tif]

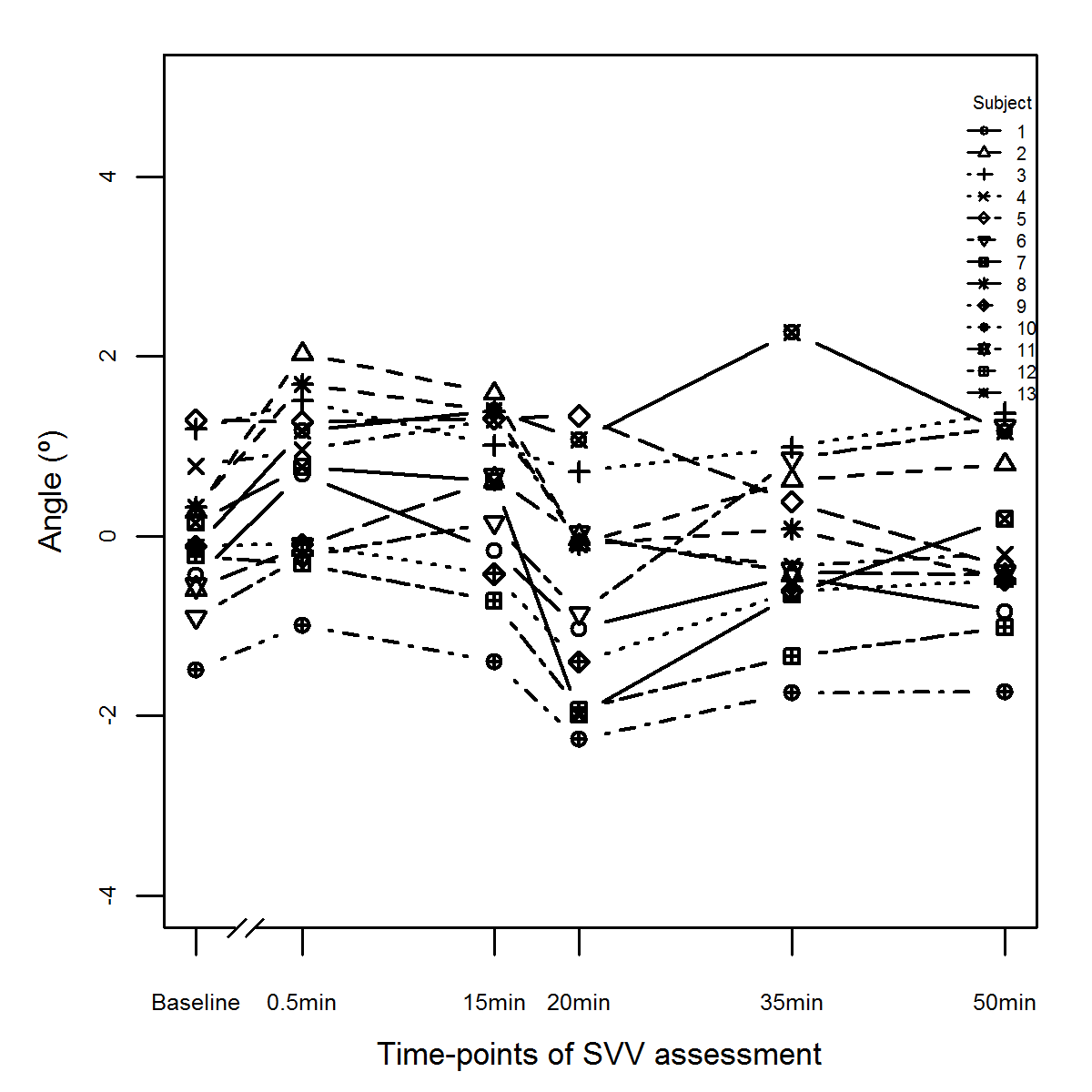

Supplement: S2 Fig — Before tDCS (T0 = Baseline), during tDCS (T1 = 0.5min: 30 seconds after the start of stimulation; T2 = 15min: 15 minutes after the beginning of stimulation); and after tDCS (T3 = 20min: immediately after; T4 = 35min: 15 minutes after the end of stimulation; T5 = 50min: 30 minutes after the stimulation). (TIF) [file pone.0152331.s002.tif]

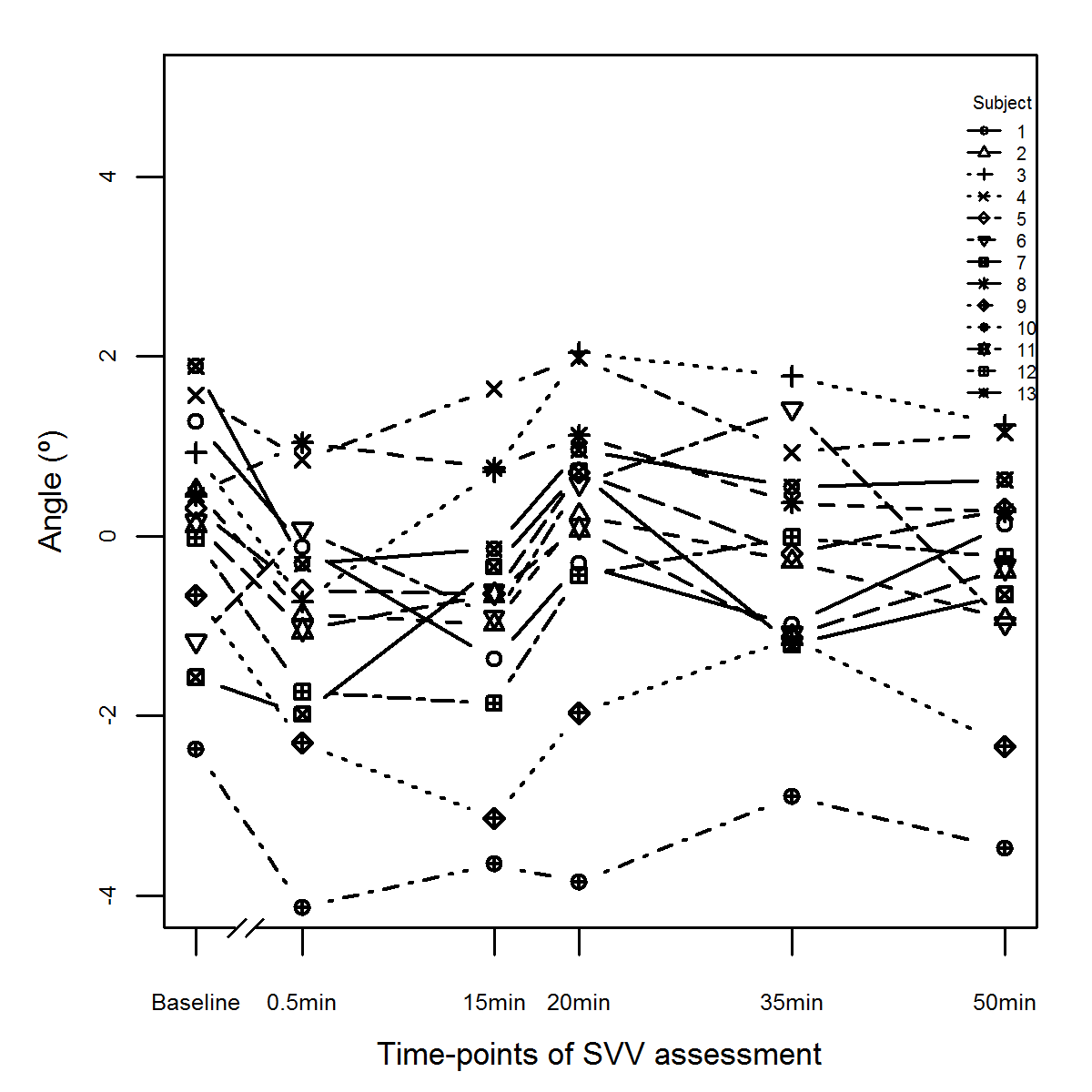

Supplement: S3 Fig — Before tDCS (T0 = Baseline), during tDCS (T1 = 0.5min: 30 seconds after the start of stimulation; T2 = 15min: 15 minutes after the beginning of stimulation); and after tDCS (T3 = 20min: immediately after; T4 = 35min: 15 minutes after the end of stimulation; T5 = 50min: 30 minutes after the stimulation). (TIF) [file pone.0152331.s003.tif]
