# Supplementary material for: Polarity-Dependent Misperception of Subjective Visual Vertical during and after Transcranial Direct Current Stimulation (tDCS)
Source: PLoS One. 2016 Mar 31;11(3):e0152331. doi: 10.1371/journal.pone.0152331 (PMC4816520; doi:10.1371/journal.pone.0152331)
Supplement: S1 Text — (PDF) [file pone.0152331.s005.pdf]

```
PROC IMPORT OUT= WORK.dados
            DATAFILE= "C:\Users\Administrador\Assessoria\Neuro
\Andamento\Taiza tdcs svv 2\dados.xls"
            DBMS=EXCEL REPLACE;
     RANGE="Plan1$";
     GETNAMES=YES;
     MIXED=NO;
     SCANTEXT=YES;
     USEDATE=YES;
     SCANTIME=YES;
RUN;

proc print;
run;

proc sort;
by Subject;
run;


proc means n mean std min q1 median q3 max maxdec=2;
class  time_categorized  tDCS Condition    ;
var svv;
run;


 proc mixed method=ml;
 class   time_categorized  tDCS Condition Subject condition_order;
 model svv =  time_categorized tDCS Condition
time_categorized*tDCS Condition condition_order/  outp=pr;
 random intercept / subject = Subject;
 lsmeans time_categorized tDCS Condition   time_categorized*tDCS
Condition condition_order;

estimate "(tDCS Condition 1 - 2)T0" tDCS Condition 1 -1 0
time_categorized*tDCS Condition 1 -1 0  0 0 0   0 0 0   0 0 0   0 0
0  0 0 0 / cl;
estimate "(tDCS Condition 1 - 3)T0" tDCS Condition 1 0 -1
time_categorized*tDCS Condition 1 0 -1  0 0 0   0 0 0   0 0 0   0 0
0  0 0 0 ;
estimate "(tDCS Condition 2 - 3)T0" tDCS Condition 0 1 -1
time_categorized*tDCS Condition 0 1 -1  0 0 0   0 0 0   0 0 0   0 0
0  0 0 0 ;

estimate "(tDCS Condition 1 - 2)T1" tDCS Condition 1 -1 0
time_categorized*tDCS Condition 0 0 0   1 -1 0   0 0 0   0 0 0   0 0
0  0 0 0 ;
estimate "(tDCS Condition 1 - 3)T1" tDCS Condition 1 0 -1
time_categorized*tDCS Condition 0 0 0   1 0 -1   0 0 0   0 0 0   0 0
0  0 0 0 ;
estimate "(tDCS Condition 2 - 3)T1" tDCS Condition 0 1 -1
time_categorized*tDCS Condition 0 0 0   0 1 -1   0 0 0   0 0 0   0 0
0  0 0 0 ;
```

```
estimate "(tDCS Condition 1 - 2)T2" tDCS Condition 1 -1 0
time_categorized*tDCS Condition 0 0 0   0 0 0  1 -1 0   0 0 0   0 0
0  0 0 0 ;
estimate "(tDCS Condition 1 - 3)T2" tDCS Condition 1 0 -1
time_categorized*tDCS Condition 0 0 0   0 0 0  1 0 -1   0 0 0   0 0
0  0 0 0 ;
estimate "(tDCS Condition 2 - 3)T2" tDCS Condition 0 1 -1
time_categorized*tDCS Condition 0 0 0   0 0 0  0 1 -1   0 0 0   0 0
0  0 0 0 ;

estimate "(tDCS Condition 1 - 2)T3" tDCS Condition 1 -1 0
time_categorized*tDCS Condition 0 0 0   0 0 0   0 0 0  1 -1 0  0 0 0
0 0 0 ;
estimate "(tDCS Condition 1 - 3)T3" tDCS Condition 1 0 -1
time_categorized*tDCS Condition 0 0 0   0 0 0   0 0 0  1 0 -1  0 0 0
0 0 0 ;
estimate "(tDCS Condition 2 - 3)T3" tDCS Condition 0 1 -1
time_categorized*tDCS Condition 0 0 0   0 0 0   0 0 0  0 1 -1  0 0 0
0 0 0 ;

estimate "(tDCS Condition 1 - 2)T4" tDCS Condition 1 -1 0
time_categorized*tDCS Condition 0 0 0   0 0 0   0 0 0  0 0 0   1 -1
0  0 0 0 ;
estimate "(tDCS Condition 1 - 3)T4" tDCS Condition 1 0 -1
time_categorized*tDCS Condition 0 0 0   0 0 0   0 0 0  0 0 0   1 0
-1  0 0 0 ;
estimate "(tDCS Condition 2 - 3)T4" tDCS Condition 0 1 -1
time_categorized*tDCS Condition 0 0 0   0 0 0   0 0 0  0 0 0   0 1
-1  0 0 0 ;

estimate "(tDCS Condition 1 - 2)T5" tDCS Condition 1 -1 0
time_categorized*tDCS Condition 0 0 0   0 0 0   0 0 0  0 0 0  0 0 0
1 -1 0 ;
estimate "(tDCS Condition 1 - 3)T5" tDCS Condition 1 0 -1
time_categorized*tDCS Condition 0 0 0   0 0 0   0 0 0  0 0 0  0 0 0
1 0 -1 ;
estimate "(tDCS Condition 2 - 3)T5" tDCS Condition 0 1 -1
time_categorized*tDCS Condition 0 0 0   0 0 0   0 0 0  0 0 0  0 0 0
0 1 -1 ;


estimate "(T0 - T1)cond 1"  time_categorized 1 -1 0 0 0 0
time_categorized*tDCS Condition 1 0 0  -1 0 0   0 0  0   0 0 0   0 0
0   0 0 0;
estimate "(T0 - T2)cond 1"  time_categorized 1 0 -1 0 0 0
time_categorized*tDCS Condition 1 0 0  0 0 0   -1 0  0   0 0 0   0 0
0   0 0 0;
estimate "(T0 - T3)cond 1"  time_categorized 1 0 0 -1 0 0
time_categorized*tDCS Condition 1 0 0  0 0 0   0 0  0   -1 0 0   0 0
0   0 0 0;
estimate "(T0 - T4)cond 1"  time_categorized 1 0 0 0 -1 0
time_categorized*tDCS Condition 1 0 0  0 0 0   0 0  0   0 0 0   -1 0
0   0 0 0;
estimate "(T0 - T5)cond 1"  time_categorized 1 0 0 0 0 -1
```

```
time_categorized*tDCS Condition 1 0 0  0 0 0   0 0  0   0 0 0   0 0
0   -1 0 0;


estimate "(T0 - T1)cond 2"  time_categorized 1 -1 0 0 0 0
time_categorized*tDCS Condition 0 1 0  0 -1 0   0 0  0   0 0 0   0 0
0   0 0 0;
estimate "(T0 - T2)cond 2"  time_categorized 1 0 -1 0 0 0
time_categorized*tDCS Condition 0 1 0  0 0 0   0 -1  0   0 0 0   0 0
0   0 0 0;
estimate "(T0 - T3)cond 2"  time_categorized 1 0 0 -1 0 0
time_categorized*tDCS Condition 0 1 0  0 0 0   0 0  0   0 -1 0   0 0
0   0 0 0;
estimate "(T0 - T4)cond 2"  time_categorized 1 0 0 0 -1 0
time_categorized*tDCS Condition 0 1 0  0 0 0   0 0  0   0 0 0   0 -1
0   0 0 0;
estimate "(T0 - T5)cond 2"  time_categorized 1 0 0 0 0 -1
time_categorized*tDCS Condition 0 1 0  0 0 0   0 0  0   0 0 0   0 0
0   0 -1 0;

estimate "(T0 - T1)cond 3"  time_categorized 1 -1 0 0 0 0
time_categorized*tDCS Condition 0 0 1  0 0 -1   0 0  0   0 0 0   0 0
0   0 0 0;
estimate "(T0 - T2)cond 3"  time_categorized 1 0 -1 0 0 0
time_categorized*tDCS Condition 0 0 1  0 0 0   0 0 -1   0 0 0   0 0
0   0 0 0;
estimate "(T0 - T3)cond 3"  time_categorized 1 0 0 -1 0 0
time_categorized*tDCS Condition 0 0 1  0 0 0   0 0  0   0 0 -1   0 0
0   0 0 0;
estimate "(T0 - T4)cond 3"  time_categorized 1 0 0 0 -1 0
time_categorized*tDCS Condition 0 0 1  0 0 0   0 0  0   0 0 0   0 0
-1   0 0 0;
estimate "(T0 - T5)cond 3"  time_categorized 1 0 0 0 0 -1
time_categorized*tDCS Condition 0 0 1  0 0 0   0 0  0   0 0 0   0 0
0   0 0 -1;


 run;


PROC CAPABILITY DATA=PR;
 HISTOGRAM RESID/NORMAL;
 QQPLOT RESID/NORMAL;
RUN;

PROC GPLOT DATA=PR;
 PLOT RESID*PRED;
RUN;
```
